# Supplementary material for: Voluntary HIV Testing and Counselling Initiatives in Occupational Settings: A Scoping Review
Source: Int J Environ Res Public Health. 2025 Feb 12;22(2):263. doi: 10.3390/ijerph22020263 (PMC11855878; doi:10.3390/ijerph22020263)
Supplement: Supplementary file 1 [file ijerph-22-00263-s001.zip › Table S2 Modified JBI data extraction tool..pdf]

**Table S2.** Modified JBI data extraction tool.

| **Data Charting Tool** | |
|---|---|
| **Item** | **Description** |
| Author(s) | |
| Year of publication | |
| Country of study | |
| Title | |
| Aim | |
| Study design | Type of study (e.g., quantitative, qualitative, mixed methods) |
| Participants | Characteristics of participants<br>• Age<br>• Gender<br>• Occupation |
| Concept | Type of voluntary HIV testing and counselling initiative or interventionIntervention description based on the 5 Item TIDiER-Lite (5 Questions)<br>• By Whom<br>• What<br>• Where<br>• To what intensity<br>• How often |
| Context | Type of occupational setting (e.g., healthcare, manufacturing, education)<br>• Public sector<br>• Private sector |
| Facilitators | Factors that promoted the implementation or uptake of the initiative/intervention |
| Barriers | Challenges encountered in implementing or engaging with the initiative/intervention |
| Key findings | Main results related to the review questions |
