# Supplementary material for: Voluntary HIV Testing and Counselling Initiatives in Occupational Settings: A Scoping Review
Source: Int J Environ Res Public Health. 2025 Feb 12;22(2):263. doi: 10.3390/ijerph22020263 (PMC11855878; doi:10.3390/ijerph22020263)
Supplement: Supplementary file 1 [file ijerph-22-00263-s001.zip › Table S3 TIDieR-Lite checklist.pdf]

Table S3. TIDieR-Lite checklist.

TIDieR-Lite Checklist Item	Description/Details
By Whom	Who delivered the intervention (profession, expertise, etc.)?
What	What was delivered during the intervention (materials, procedures, etc.)?
Where	Where was the intervention delivered (location, setting)?
To What Intensity	What was the intensity or dose of the intervention?
How Often	How frequently was the intervention delivered?
