# Supplementary material for: Voluntary HIV Testing and Counselling Initiatives in Occupational Settings: A Scoping Review
Source: Int J Environ Res Public Health. 2025 Feb 12;22(2):263. doi: 10.3390/ijerph22020263 (PMC11855878; doi:10.3390/ijerph22020263)
Supplement: Supplementary file 1 [file ijerph-22-00263-s001.zip › Text S1 Search strategy.pdf]

Text S1. Search strategy for all databases.

	**Search on MEDLINE through OVID interface (29/10/23)**	**Results**
1	exp HIV Testing/	7698
2	((HIV or AIDS or HIV-AIDS) adj4 (test* or diagnos* or counsel?ing or advice or guidance or care or prevention)).mp.	87973
3	((HIV or AIDS or HIV-AIDS) adj5 (program* or intervention* or initiative* or policy or policies or promot* or campaign* or activit*)).mp.	45042
4	1 or 2 or 3	115194
5	exp Workplace/	30060
6	(workplace* or worksite* or employee* or worker* or office* or company or companies or factory or factories or organi#ation* or institution*).mp.	1838648
7	((work* or employ* or occupational) adj3 (place* or site* or location* or setting* or environment*)).mp.	68588
8	5 or 6 or 7	1872650
9	4 and 8	21557
10	(voluntary or opt-in or "HIV test").mp.	88619
11	9 and 10	**1365**

	**Search on EMBASE through OVID interface (29/10/23)**	**Results**
1	exp HIV Testing/	16074
2	((HIV or AIDS or HIV-AIDS) adj4 (test* or diagnos* or counsel?ing or advice or guidance or care or prevention)).mp.	116833
3	((HIV or AIDS or HIV-AIDS) adj5 (program* or intervention* or initiative* or policy or policies or promot* or campaign* or activit*)).mp.	53946

4	1 or 2 or 3	147061
5	exp Workplace/	54966
6	(workplace* or worksite* or employee* or worker* or office* or company or companies or factory or factories or organi#ation* or institution*).mp.	2539126
7	((work* or employ* or occupational) adj3 (place* or site* or location* or setting* or environment*)).mp.	108734
8	5 or 6 or 7	2592566
9	4 and 8	24593
10	(voluntary or opt-in or "HIV test").mp.	121746
11	9 and 10	**4068**

	**Search on CINAHL Ultimate through EBSCOhost interface (19/11/23)**	**Results**
1	hiv or aids or acquired human immunodeficiency syndrome or human immunodeficiency virus	146,193
2	test* or diagnos* or counsel?ing or advice or guidance or care or prevention	3,586,617
3	program* or intervention* or initiative* or policy or policies or promot* or campaign* or activit*	1,702,039
4	1 and 2 and 3	37,650
5	Workplace	51,888
6	workplace* or worksite* or employee* or worker* or office* or company or companies or factory or factories or organi#ation* or institution*	816,654
7	work* or employ* or occupational) adj3 (place* or site* or location* or setting* or environment*	1,495,485
8	5 or 6 or 7	1,934,339
9	4 and 8	17,255

10	voluntary* or opt-in*	25,065
11	9 and 10	**572**

	**Search on Scopus (22/11/23)**	**Results**
1	TITLE-ABS-KEY ("hiv" OR "AIDS" OR "acquired human immunodeficiency syndrome" OR "human immunodeficiency virus")	729,288
2	TITLE-ABS-KEY (test* or diagnos* or counsel?ing or advice or guidance or care or prevention)	20,827,714
3	TITLE-ABS-KEY (program* or intervention* or initiative* or policy or policies or promot* or campaign* or activit*)	16,481,055
4	1 and 2 and 3	126,199
5	TITLE-ABS-KEY (workplace)	178,082
6	TITLE-ABS-KEY (workplace* OR worksite* OR employee* OR worker* OR office* OR company OR companies OR factory OR factories OR organi#ation* OR institution*)	3,492,685
7	TITLE-ABS-KEY ("workplace" OR "work environment" OR "work site" OR "employ* site" OR "occupational place" OR "occupational site" OR "work location")	72,288
8	5 or 6 or 7	3,525,201
9	4 and 8	15,929
10	TITLE-ABS-KEY (voluntary* OR opt-in*)	161,680
11	9 and 10	**543**

	**Search on Epistemonikos (22/11/23)**	**Results**

1	(title:("hiv" OR "AIDS" OR "acquired human immunodeficiency syndrome" OR "human immunodeficiency virus") OR abstract:("hiv" OR "AIDS" OR "acquired human immunodeficiency syndrome" OR "human immunodeficiency virus")) AND (title:(test* OR diagnos* OR counsel?ing OR advice OR guidance OR care OR prevention) OR abstract:(test* OR diagnos* OR counsel?ing OR advice OR guidance OR care OR prevention)) AND (title:(program* OR intervention* OR initiative* OR policy OR policies OR promot* OR campaign* OR activit*) OR abstract:(program* OR intervention* OR initiative* OR policy OR policies OR promot* OR campaign* OR activit*))	43,272
2	(title:(workplace* OR worksite* OR employee* OR worker* OR office* OR company OR companies OR factory OR factories OR organi#ation* OR institution*) OR abstract:(workplace* OR worksite* OR employee* OR worker* OR office* OR company OR companies OR factory OR factories OR organi#ation* OR institution*)) OR (title:("work place" OR "work environment" OR "work site" OR "employ* site" OR "occupational place" OR "occupational site" OR "work location") OR abstract:("work place" OR "work environment" OR "work site" OR "employ* site" OR "occupational place" OR "occupational site" OR "work location"))	182,502
3	1 and 2	6,044
4	(title:(voluntary* OR opt-in*) OR abstract:(voluntary* OR opt-in*))	14,787
5	3 and 4	**250**

Google Scholar (22/11/23)

(hiv OR aids OR acquired human immunodeficiency syndrome OR human immunodeficiency virus) AND (test* OR diagnos* OR counsel?ing OR advice OR intervention OR initiative*) AND (workplace*

OR worksite* OR employee* OR worker* OR company OR organi#ation* OR institution*) AND (voluntary* OR opt-in*) **100**

The Cochrane Register of Control Trials (22/11/23)

80 Trials matching "hiv" OR "AIDS" OR "acquired human immunodeficiency syndrome" OR "human immunodeficiency virus" in Title Abstract Keyword AND test* OR diagnos* OR counsel?ing OR advice OR guidance OR care OR prevention OR program* OR intervention* OR initiative* OR policy OR policies OR promot* OR campaign* OR activit* in Title Abstract Keyword AND workplace* OR worksite* OR employee* OR worker* OR office* OR company OR companies OR factory OR factories OR organi#ation* OR institution* in Title Abstract Keyword AND voluntary* OR opt-in* in Title Abstract Keyword
