# Supplementary material for: Voluntary HIV Testing and Counselling Initiatives in Occupational Settings: A Scoping Review
Source: Int J Environ Res Public Health. 2025 Feb 12;22(2):263. doi: 10.3390/ijerph22020263 (PMC11855878; doi:10.3390/ijerph22020263)
Supplement: Supplementary file 1 [file ijerph-22-00263-s001.zip › Text S2 Excluded studies.pdf]

Text S2. Excluded Studies

Non-relevance to workplace HIV testing and counselling (39 articles)

1- Shapiro AE, van Heerden A, Krows M, Sausi K, Sithole N, Schaafsma TT, Koole O, van Rooyen H, Celum CL, Barnabas RV. An implementation study of oral and blood-based HIV self-testing and linkage to care among men in rural and peri-urban KwaZulu-Natal, South Africa. Journal of the International AIDS Society. 2020 Jun;23: e25514.HIV/AIDS, Crime and Small Business in South Africa.

2- Isaacs E, Friedrich C. HIV/AIDS, crime and small business in South Africa. In Developmental entrepreneurship: adversity, risk, and isolation 2006 Aug 22 (pp. 167-183). Emerald Group Publishing Limited.

3- Mamulwar M, Prasad VS, Nirmalkar A, Goli S, Jadhav S, Kumbhar S, Kale V, Michael E, Ford TM, Nazarov D, Honig L. Community-based point-of-care testing to identify new HIV infections: A cross-sectional study from Pune, India. Medicine. 2021 Nov 19;100(46): e27817.

4- Abeid R, Mergenthaler C, Muzuka V, Goodluck A, Nkwabi T, Bigio J, Vasquez N A, Pande T, Haraka F, Creswell J, Rahman T. Increasing TB/HIV Case Notification through an Active Case-Finding Approach among Rural and Mining Communities in Northwest Tanzania. Journal of Tropical Medicine. 2022;2022(1):4716151.

5- Khumalo-Sakutukwa G, Morin SF, Fritz K, Charlebois ED, Van Rooyen H, Chingono A, Modiba P, Mrumbi K, Visrutaratna S, Singh B, Sweat M. Project Accept (HPTN 043): a community-based intervention to reduce HIV incidence in populations at risk for HIV in sub-Saharan Africa and Thailand. JAIDS Journal of Acquired Immune Deficiency Syndromes. 2008 Dec 1;49(4):422-31."Amare con Sapienza": An Italian digital ambient media prevention campaign in one of the largest European University to increase U=U, PreP, TasP knowledge

6- Vita S, Lichtner M, Morlino G, Celani L, Zingaropoli MA, Perri V, Valeri S, Dezza FC, Santinelli L, Masci G, Falciano M. " Amare con Sapienza": an Italian digital ambient media prevention

campaign in one of the largest European University to increase U= U, PreP, TasP knowledge. InHIV MEDICINE 2019 Nov 1 (Vol. 20, pp. 102-102). 111 RIVER ST, HOBOKEN 07030-5774, NJ USA: WILEY.

7- Makinde OA. Community-based HIV counselling and testing as a means of improving HIV programme performance. The Lancet Global Health. 2014 Sep 1;2(9): e505.

8- Marum E, Taegtmeyer M, Chebet K. Scale-up of voluntary HIV counseling and testing in Kenya. Jama. 2006 Aug 16;296(7):859-62.

9- Were W, Mermin J, Bunnell R, Ekwaru JP, Kaharuza F. Home-based model for HIV voluntary counselling and testing. Lancet. 2003 May 3;361(9368):1569.

10- James AJ, Marable D, Cubbison CV, Tarbox AA, Mejia DL, Oo SA, Freedberg KA, Levison JH. HIV testing in a large community health center serving a multi-cultural patient population: a qualitative study of providers. AIDS care. 2019 Dec 2;31(12):1585-92.

11- Ron Levey I, Wang W. Unravelling the quality of HIV counselling and testing services in the private and public sectors in Zambia. Health policy and planning. 2014 Jul 1;29(suppl_1): i30-7.

12- Wainberg MA, Hull MW, Girard PM, Montaner JS. Achieving the 90–90–90 target: incentives for HIV testing. The Lancet Infectious Diseases. 2016 Nov 1;16(11):1215-6.

13- Nokes K, Johnson MO, Webel A, Rose CD, Phillips JC, Sullivan K, Tyer-Viola L, Rivero-Méndez M, Nicholas P, Kemppainen J, Sefcik E. Focus on increasing treatment self-efficacy to improve human immunodeficiency virus treatment adherence. Journal of Nursing Scholarship. 2012 Dec;44(4):403-10.

14- Mayer KH, Powderly WG, Mayer KH. Centers for Disease Control and Prevention revised guidelines for human immunodeficiency virus (HIV) counseling, testing, and referral: targeting HIV specialists. Clinical infectious diseases. 2003 Sep 15;37(6):813-9.

15- Fernàndez-Lopez L, Rifà B, Pujol F, Becerra J, Pérez M, Meroño M, Zaragoza K, Rafel A, Diaz O, Avellaneda A, Casado MJ. Impact of the introduction of rapid HIV testing in the Voluntary

Counselling and Testing sites network of Catalonia, Spain. International journal of STD & AIDS. 2010 Jun;21(6):388-91.

16- De Cock KM, El-Sadr WM. From START to finish implications of the START study. The Lancet Infectious Diseases. 2016 Jan 1;16(1):13-4.

17- Woods WJ, Erwin K, Lazarus M, Serice H, Grinstead O, Binson D. Building stakeholder partnerships for an on-site HIV testing programme. Culture, health & sexuality. 2008 Apr 1;10(3):249-62.

18- Majam M. Progress on the scaling up of HIV testing in South Africa through varied distribution models using the oral HIV self-test kit. Oral diseases. 2020 Sep; 26:137-40.

19- Lewis NM, Gahagan JC, Stein C. Preferences for rapid point-of-care HIV testing in Nova Scotia, Canada. Sexual health. 2013 Feb 1;10(2):124-32.

20- Galea JT, Puma D, Tzelios C, Valdivia H, Millones AK, Jiménez J, Brooks MB, Yuen CM, Lecca L, Becerra MC, Keshavjee S. A structured community engagement strategy to support uptake of TB active case-finding. Public Health Action. 2022 Mar 21;12(1):18-23.

21- Khamisa N, Mokgobi M. Risky sexual behaviour and human immunodeficiency virus (HIV) and acquired immune deficiency syndrome (AIDS) among healthcare workers. Southern African Journal of HIV Medicine. 2018 Jan 1;19(1):1-5.

22- Bokhour B, Henry SR, Saifu H, Fix GM, Fletcher M, Goetz MB, Knapp H, Burgess J, Asch S. barriers and facilitators to implementing a multimodal intervention to increase hiv testing. in journal of general internal medicine 2012 jul 1 (vol. 27, pp. s131-s131). 233 Spring st, New York, NY 10013 USA: Springer.

23- Rutenberg N, Tun W, Borse NN. Lessons learned and study results from HIVCore: an HIV implementation science initiative. African Journal of Reproduction and Gynaecological Endoscopy. 2016 Jul 1;19.

24- Babatunde OT, Babatunde LS, Oyedeji OA, Owa IA. Acceptance of Human Immunodeficiency Virus Testing among Caregivers of Children using Provider-Initiated Testing and Counselling

Strategy in Ido-ekiti, Nigeria: A Cross-sectional Study. Journal of Clinical & Diagnostic Research. 2019 Aug 1;13(8).

25- Mosoko JJ, Macauley IB, Zoungkanyi AC, Bella A, Koulla-Shiro S. Human immunodeficiency virus infection and associated factors among specific population subgroups in Cameroon. AIDS and Behavior. 2009 Apr; 13:277-87.

26- Rayment M, Thornton A, Mandalia S, Elam G, Atkins M, Jones R, Nardone A, Roberts P, Tenant-Flowers M, Anderson J, Ann K. Sullivan on behalf of the HINTS Study Group. HIV testing in non-traditional settings–the HINTS study: a multi-centre observational study of feasibility and acceptability. PloS one. 2012 Jun 22;7(6): e39530.

27- Venter F, Majam M, Jankelowitz L, Adams S, Moorhouse M, Carmona S, Stevens W, Msimanga BR, Allen D, Balani P, Nevhutalu Z. South African HIV self-testing policy and guidance considerations. Southern African journal of HIV medicine. 2017;18(1).

28- Anderson TN, Louw-Potgieter J. An implementation evaluation of a voluntary counselling and testing programme for the human immunodeficiency virus (HIV) and acquired immunodeficiency syndrome (AIDS). SA Journal of Industrial Psychology. 2012 Jan;38(1):1-0.

29- Muwanguzi PA, Nelson LE, Ngabirano TD, Kiwanuka N, Osingada CP, Sewankambo NK. Linkage to care and treatment among men with reactive HIV self-tests after workplace-based testing in Uganda: a qualitative study. Frontiers in public health. 2022 Oct 12; 10:650719.

30- Winkelmann M, Sorrentino JN, Klein M, Macke C, Mommsen P, Brand S, Schröter C, Krettek C, Zeckey C. Is there a benefit for health care workers in testing HIV, HCV and HBV in routine before elective arthroplasty? Orthopaedics & Traumatology: Surgery & Research. 2016 Jun 1;102(4):513-6.

31- Ishimaru T, Wada K, Smith DR. HIV testing and attitudes among the working-age population of Japan: annual health checkups may offer an effective way forward. Industrial health. 2016;54(2):116-22.

32- Mizuki K, Ishimaru T, Imahashi M, Ikushima Y, Takahashi H, Masuda M, Yokomaku Y. Workplace factors associated with willingness to undergo human immunodeficiency virus testing during workplace health checkups. Environmental Health and Preventive Medicine. 2023; 28:52.

33- Nabukalu D, Ponticiello M, Bennett T, Clark S, King R, Mwanga-Amumpaire J, Sundararajan R. Factors associated with HIV testing among traditional healers and their clients in rural Uganda: results from a cross-sectional study. International journal of STD & AIDS. 2021 Oct;32(11):1043-51.

34- Munyati SS, Redzo N, Dauya E, Matambo R, Makamure B, Bandason T, Butterworth AE, Gwanzura L, Rusakaniko S, Mason PR, Corbett EL. Human immunodeficiency virus, smoking and self-rated health in Harare, Zimbabwe. The international Journal of Tuberculosis and Lung Disease. 2006 Nov 1;10(11):1279-85.

35- Fultz E, Francis JM. Employer-sponsored programmes for the prevention and treatment of HIV/AIDS: Recent experience from sub-Saharan Africa. International Social Security Review. 2011 Jul;64(3):1-9.

36- Berkley-Patton J, Thompson CB, Goggin K, Catley D, Berman M, Bradley-Ewing A, Derose KP, Resnicow K, Allsworth J, Simon S. A religiously tailored, multilevel intervention in African American churches to increase HIV testing: Rationale and design of the Taking It to the Pews cluster randomized trial. Contemporary Clinical Trials. 2019 Nov 1; 86:105848.

37- Strauss M, George G, Lansdell E, Mantell JE, Govender K, Romo M, Odhiambo J, Mwai E, Nyaga EN, Kelvin EA. HIV testing preferences among long distance truck drivers in Kenya: a discrete choice experiment. AIDS care. 2018 Jan 2;30(1):72-80.

38- Kiwanuka N, Ssetaala A, Mpendo J, Wambuzi M, Nanvubya A, Sigirenda S, Nalutaaya A, Kato P, Nielsen L, Kaleebu P, Nalusiba J. High HIV-1 prevalence, risk behaviours, and willingness to participate in HIV vaccine trials in fishing communities on Lake Victoria, Uganda. Journal of the International AIDS Society. 2013 Jan;16(1):18621.

39- Vass JR. The role of HIV/AIDS committees in effective workplace governance of HIV/AIDS in South African small and medium-sized enterprises (SMEs). SAHARA-J: Journal of Social Aspects of HIV/AIDS. 2008 Jul 14;5(1):2-10.

Articles without assessing HIV testing and counselling intervention (28 articles)

1- Zhou W, Deng W, Luo J, Bai Y, He Z, Wang H. Predictors for HIV testing among Chinese workers in infrastructure construction enterprises in Kenya. BMC Public Health. 2021 Dec; 21:1-8.

2- Rothberg A, Van Huyssteen K. Employees' perceptions of the Aid-for-AIDS disease-management programme, South Africa. African journal of AIDS research. 2008 Nov 1;7(3):335-9.

3- Estcourt C, Saunders J, Mercer C, Sutcliffe L, Hart G. P5-S7. 01 Exploring the acceptability of medical, educational and sport settings for STI screening: stratified random probability survey of young men in the UK. Sexually Transmitted Infections. 2011 Jul 1;87(Suppl 1): A342.

4- Nardell MF, Govathson C, Mngadi-Ncube S, Ngcobo N, Letswalo D, Lurie M, Miot J, Long L, Katz IT, Pascoe S. Migrant men and HIV care engagement in Johannesburg, South Africa. BMC Public Health. 2024 Feb 12;24(1):435.

5- Ford K, Holumyong C. HIV testing and cross border migrant vulnerability: social integration and legal/economic status among cross border migrant workers in Thailand. AIDS and Behavior. 2016 Apr; 20:919-27.

6- Ishimaru T, Imahashi M, Ikushima Y, Takahashi H, Yokomaku Y. Need for and barriers to voluntary HIV testing during health checkups in Japanese companies. Safety and Health at Work. 2022 Jan 1;13: S108-9.

7- Kinney S, Lea CS, Kearney G, Kinsey A, Amaya C. Predictors for using a HIV self-test among migrant and seasonal farmworkers in North Carolina. International Journal of Environmental Research and Public Health. 2015 Jul;12(7):8348-58.

8- Tshuma N, Muloongo K, Setswe G, Chimoyi L, Sarfo B, Burger D, Nyasulu PS. Potential barriers to rapid testing for human immunodeficiency virus among a commuter population in Johannesburg, South Africa. HIV/AIDS-Research and Palliative Care. 2014 Dec 30:11-9.

9- Kirakoya-Samadoulougou F, Yaro S, Deccache A, Fao P, Defer MC, Meda N, Robert A, Nagot N. Voluntary HIV testing and risky sexual behaviours among health care workers: a survey in rural and urban Burkina Faso. BMC public health. 2013 Dec; 13:1-7.

10- Lau JT, Tsui HY, Cheng S, Pang M. A randomized controlled trial to evaluate the relative efficacy of adding voluntary counseling and testing (VCT) to information dissemination in reducing HIV-related risk behaviors among Hong Kong male cross-border truck drivers. AIDS care. 2010 Jan 1;22(1):17-28.

11- Middleton M, Somerset S, Evans C, Blake H. Test@ Work texts: mobile phone messaging to increase awareness of HIV and HIV testing in UK construction employees during the COVID-19 pandemic. International journal of environmental research and public health. 2020 Nov;17(21):7819.

12- Knežević B, Zahariev Vukšinić K, Šijaković A. the prevention of hiv/aids infection among the migrant workers population. Sigurnost: časopis za sigurnost u radnoj i životnoj okolini. 2012 Dec 18;54(4):383-8.

13- Davis A, Terlikbayeva A, Terloyeva D, Primbetova S, El-Bassel N. What prevents central Asian migrant workers from accessing HIV testing? Implications for increasing HIV testing uptake in Kazakhstan. AIDS and Behavior. 2017 Aug; 21:2372-80.

14- Hamill M, Copas A, Murphy SM. Incentives for voluntary HIV testing in NHS staff. Occupational Medicine. 2006 Sep 1;56(6):426-9.

15- Soko D, Umar E, Noniwa T, Lakudzala A. HIV and AIDS workplace interventions; Gaps between policy and practice at the College of Medicine. Malawi Medical Journal. 2012;24(3):52-5.

16- Chamratrithirong A, Ford K, Punpuing S, Prasartkul P. A workplace intervention program and the increase in HIV knowledge, perceived accessibility and use of condoms among young factory workers in Thailand. SAHARA-J: Journal of Social Aspects of HIV/AIDS. 2017;14(1):132-9.

17- Sasaki Y, Arifin A, Ali M, Kakimoto K. Willingness to undergo HIV testing among factory workers in Surabaya, Indonesia. AIDS care. 2011 Oct 1;23(10):1305-13.

18- Blake H, Somerset S, Evans C. Development and Fidelity testing of the test@ work digital toolkit for employers on workplace health checks and opt-in HIV testing. International journal of environmental research and public health. 2020 Jan;17(1):379.

19- Ha T, Shi H, Givens D, Nguyen T, Nguyen N. Factors impacting HIV testing among young sexually active women migrant workers in Vietnamese industrial zones. BMC Public Health. 2023 Oct 6;23(1):1938.

20- Musumari PM, Chamchan C. Correlates of HIV testing experience among migrant workers from Myanmar residing in Thailand: a secondary data analysis. PloS one. 2016 May 3;11(5): e0154669.

21- Dickinson D, Stevens M. Understanding the response of large South African companies to HIV/AIDS. SAHARA-J: Journal of Social Aspects of HIV/AIDS. 2005 Jul 1;2(2):286-95.

22- Zungu LI, Sanni BA. Acceptance and uptake of voluntary HIV testing among healthcare workers in a South African public hospital. South African Family Practice. 2011 Sep 1;53(5):488-94.

23- Ssekankya V, Githaiga SK, Aleko T, Munguciada EF, Nabakka VP, Kyalisiima JJ, Ndyabakira A, Migisha R. Factors Influencing Utilization of HIV Testing Services among Boda-Boda Riders in Kabarole District, Southwestern Uganda: A Cross-Sectional Study. BioMed Research International. 2021;2021(1):8877402.

24- Khan R, Yassi A, Engelbrecht MC, Nophale L, van Rensburg AJ, Spiegel J. Barriers to HIV counselling and testing uptake by health workers in three public hospitals in Free State Province, South Africa. AIDS care. 2015 Feb 1;27(2):198-205.

25- Day JH, Miyamura K, Grant AD, Leeuw A, Munsamy J, Baggaley R, Churchyard GJ. Attitudes to HIV voluntary counselling and testing among mineworkers in South Africa: will availability of antiretroviral therapy encourage testing? AIDS care. 2003 Oct 1;15(5):665-72.

26- Betunga B, Atuhaire P, Nakasiita C, Kanyamuneza C, Namiiro P, Tugume J, Hairat M, Sarki AM, Mugabi B, Lilian B, Mugisha R. Factors influencing the use of multiple HIV prevention services among transport workers in a city in southwestern Uganda. PLOS Global Public Health. 2023 Mar 2;3(3): e0001350.

27- Blake H, Banerjee A, Evans C. Employer attitudes towards general health checks and HIV testing in the workplace. Public Health. 2018 Mar 1; 156:34-43.

28- Knoblauch AM, Divall MJ, Owuor M, Nduna K, Ng'uni H, Musunka G, Pascall A, Utzinger J, Winkler MS. Experience and lessons from health impact assessment guiding prevention and control of HIV/AIDS in a copper mine project, northwestern Zambia. Infectious diseases of poverty. 2017 Aug 1;6(04):22-32.

Relevance to sex workers (24 articles)

1- Tokar A, Osborne J, Slobodianiuk K, Essink D, Lazarus JV, Broerse JE. 'Virus Carriers' and HIV testing: navigating Ukraine's HIV policies and programming for female sex workers. Health research policy and systems. 2019 Dec; 17:1-20.

2- Mee P, Neuman M, Kumwenda M, Sambo M, Lora W, Indravudh PP, Hatzold K, Johnson C, Corbett EL, Desmond N. Does the use of HIV self-testing kits lead to unintended effects? Evidence from female sex workers in Malawi. injournal of the international aids' society 2019 jul 1 (vol. 22, pp. 54-54). The Atrium, Southern Gate, Chichester po19 8sq, W Sussex, England: John Wiley & Sons Ltd.

3- Huang Y, Zhang Y, Li K, Zhao J. Changes in prevalence of HIV or syphilis among male sex workers and non-commercial men who have sex with men in Shenzhen, China: results of a second survey. PLoS One. 2016 Dec 9;11(12): e0167619.

4- Grosso AL, Ketende S, Dam K, Papworth E, Ouedraogo HG, Ky-Zerbo O, Baral S. Structural determinants of health among women who started selling sex as minors in Burkina Faso. JAIDS Journal of Acquired Immune Deficiency Syndromes. 2015 Mar 1;68: S162-70.

5- Nyanzi S. Homosexuality, sex work, and HIV/AIDS in displacement and post-conflict settings: the case of refugees in Uganda. International Peacekeeping. 2013 Aug 1;20(4):450-68.

6- Xun H, Kang D, Huang T, Qian Y, Li X, Wilson EC, Yang S, Jiang Z, Gong C, Tao X, Zhang X. Factors associated with willingness to accept oral fluid HIV rapid testing among most-at-risk populations in China. PloS one. 2013 Nov 19;8(11): e80594.

7- Charles B, Jeyaseelan L, Edwin Sam A, Kumar Pandian A, Thenmozhi M, Jeyaseelan V. Trends in risk behaviors among female sex workers in south India: Priorities for sustaining the reversal of HIV epidemic. AIDS care. 2013 Sep 1;25(9):1129-37.

8- Chabata ST, Hensen B, Chiyaka T, Mushati P, Musemburi S, Dirawo J, Busza J, Floyd S, Birdthistle I, Hargreaves JR, Cowan FM. The impact of the DREAMS partnership on HIV incidence among young women who sell sex in two Zimbabwean cities: results of a non-randomised study. BMJ Global Health. 2021 Apr 1;6(4): e003892.

9- Mizinduko M, Moen K, Pinkowski Tersbøl B, Likindikoki SL, Alexander Ishungisa M, Leyna GH, Makyao N, Leshabari MT, Ramadhani A, Wolf Meyrowitsch D, Lange T. HIV testing and associated factors among female sex workers in Tanzania: approaching the first 90% target? AIDS care. 2023 Jun 3;35(6):850-8.

10- Agot K, Muthumbi G, Kimani J, Gichangi P, Musyoki H, Onyango J, Mando R, Odonde P, Owino C, Mukiri E, Kioo C. Potential Benefits and Risks of HIV Self-testing Access by Female Sex Workers: Views of Service Providers, Outreach Workers and Sex Workers in Kenya. In

AIDS Research And Human Retroviruses 2016 Oct 1 (Vol. 32, Pp. 164-164). 140 Huguenot Street, 3rd Fl, New Rochelle, Ny 10801 Usa: Mary Ann Liebert, Inc.

11- Schwartz SR, Papworth E, Ky-Zerbo O, Anato S, Grosso A, Ouedraogo HG, Ketende S, Pitche VP, Baral S. Safer conception needs for HIV prevention among female sex workers in Burkina Faso and Togo. Infectious Diseases in Obstetrics and Gynecology. 2014;2014(1):296245.

12- Jana S, Ray P, Roy S, Kadam A, Gangakhedkar RR, Rewari BB, Moses S, Becker ML. Successful integration of HIV pre-exposure prophylaxis into a community-based HIV prevention program for female sex workers in Kolkata, India. International journal of STD & AIDS. 2021 Jun;32(7):638-47.

13- Rice B, Machingura F, Maringwa G, Magutshwa S, Kujeke T, Jamali G, Busza J, de Wit M, Fearon E, Hanisch D, Yekeye R. Adolescent girls who sell sex in Zimbabwe: HIV risk, behaviours and service engagement. JAIDS Journal of Acquired Immune Deficiency Syndromes. 2022 May 13:10-97.

14- Tokar A, Sazonova I, Mishra S, Smyrnov P, Saliuk T, Lazarus JV, Broerse JE, Roura M, Blanchard J, Becker ML. HIV testing behaviour and HIV prevalence among female sex workers in Ukraine: findings from an Integrated Bio-Behavioural Survey, 2013–2014. Sexually Transmitted Infections. 2019 May 1;95(3):193-200.

15- Vandepitte J, Bukenya J, Weiss HA, Nakubulwa S, Francis SC, Hughes P, Hayes R, Grosskurth H. HIV and other sexually transmitted infections in a cohort of women involved in high-risk sexual behavior in Kampala, Uganda. Sexually transmitted diseases. 2011 Apr 1;38(4):316-23.

16- Nguyen VT, Phan HT, Kato M, Nguyen QT, Le Ai KA, Vo SH, Thanh DC, Baggaley RC, Johnson CC. Community-led HIV testing services including HIV self-testing and assisted partner notification services in Vietnam: lessons from a pilot study in a concentrated epidemic setting. Journal of the International AIDS Society. 2019 Jul;22: e25301.

17- Uddin SM, Hossain MG, Islam MA, Islam MN, Aik S, Kamarul T. High-risk behavior of HIV/AIDS among female sex workers in Bangladesh: survey in Rajshahi City. Japanese journal of infectious diseases. 2014;67(3):191-6.

18- Lahuerta M, Torrens M, Sabidó M, Batres A, Casabona J. Sexual risk behaviours and barriers to HIV testing among clients of female sex workers in Guatemala: a qualitative study. Culture, health & sexuality. 2013 Jul 29;15(7):759-73.

19- Netak Y, Vasylyev M, Sluzhynska O, Sluzhynska M, Rybak N, Ostapuyk L, Grushynska O, Krystynyak O. Results of STI/HIV Counseling and Testing Program for Commercial Sex Workers, Drug Users, Men Have Sex with Men and Former Prisoners During 2012-2013 in Lviv Region (Ukraine). In Sexually Transmitted Diseases 2014 Jun 1 (vol. 41, pp. s123-s123). Two Commerce SQ, 2001 Market ST, Philadelphia, PA 19103 USA: Lippincott Williams & Wilkins.

20- Fang Y, Zhang Y, Wang Z, Ip M, Li J, Lau JT. Low uptake of HIV testing among male clients of female sex workers in China. AIDS care. 2019 Feb 1;31(2):193-8.

21- Ortblad K, Kibuuka Musoke D, Ngabirano T, Nakitende A, Magoola J, Kayiira P, Taasi G, Barresi LG, Haberer JE, McConnell MA, Oldenburg CE. Direct provision versus facility collection of HIV self-tests among female sex workers in Uganda: a cluster-randomized controlled health systems trial. PLoS medicine. 2017 Nov 28;14(11): e1002458.

22- Conserve DF, Issango J, Kilale AM, Njau B, Nhigula P, Memiah P, Mbita G, Choko AT, Hamilton A, King G. Developing national strategies for reaching men with HIV testing services in Tanzania: results from the male catch-up plan. BMC health services research. 2019 Dec; 19:1-0.

23- Diserens EA, Bodenmann P, N'Garambe C, Ansermet-Pagot A, Vannotti M, Masserey E, Cavassini M. Clients of sex workers in Switzerland: it makes sense to counsel and propose rapid test for HIV on the street, a preliminary report. BMC infectious diseases. 2010 Dec; 10:1-5.

24- Woodford MR, Chakrapani V, Newman PA, Shunmugam M. Barriers and facilitators to voluntary HIV testing uptake among communities at high risk of HIV exposure in Chennai, India. Global public health. 2016 Mar 15;11(3):363-79.

Addressing blood fluid exposure (6 articles)

1- Adal O, Abebe A. HIV Sero-Status of Health Care Workers in Addis Ababa Public Hospitals After Post-Exposure Blood and Body Fluids: A Cross-Sectional Study, October 2022. Clinical Medicine Insights: Case Reports. 2023 Mar; 16:11795476231161406.

2- Landrum ML, Wilson CH, Perii LP, Hannibal SL, O'Connell RJ. Usefulness of a rapid human immunodeficiency virus-1 antibody test for the management of occupational exposure to blood and body fluid. Infection Control & Hospital Epidemiology. 2005 Sep;26(9):768-74.

3- Gerberding JL. Occupational exposure to HIV in health care settings. New England Journal of Medicine. 2003 Feb 27;348(9):826-33.

4- Chalya PL, Seni J, Mushi MF, Mirambo MM, Jaka H, Rambau PF, Kapesa A, Ngallaba SE, Massinde AN, Kalluvya SE. Needle-stick injuries and splash exposures among health-care workers at a tertiary care hospital in north-western Tanzania. Tanzania Journal of Health Research. 2015 Apr 4;17(2).

5- Weber D, Wang SA, Panlilio AL, Doi PA, White AD, Stek M, Saah A. Experience of healthcare workers taking postexposure prophylaxis after occupational HIV exposures: findings of the HIV Postexposure Prophylaxis Registry. Infection Control & Hospital Epidemiology. 2000 Dec;21(12):780-5.

6- Venier AG, Vincent A, L'heriteau F, Floret N, Senechal H, Abiteboul D, Reyreaud E, Coignard B, Parneix P. Surveillance of occupational blood and body fluid exposures among French healthcare workers in 2004. Infection Control & Hospital Epidemiology. 2007 Oct;28(10):1196-201.

Discussing HIV prevalence in workplaces (6 articles)

1- Semá Baltazar C, DeLima YV, Ricardo H, Botão C, Chitsondzo Langa D, Da Costa P, Malamule D, Augusto Â, Viegas S, Obisie-Nmehielle N, Tomm-Bonde L. HIV prevalence and TB in migrant miners' communities of origin in Gaza Province, Mozambique: The need for increasing awareness and knowledge. Plos one. 2020 Apr 8;15(4): e0231303.

2- Bozicevic I, Guezzar F, Stulhofer A, Bennani A, Handanagic S, Barbaric J, El Rhilani H, Alami K, Khattabi H, Riedner G, Maaroufi A. HIV prevalence and related risk behaviours in female seasonal farm workers in Souss Massa Draa, Morocco: results from a cross-sectional survey using cluster-based sampling. Sexually transmitted infections. 2018 Nov 1;94(7):515-7.

3- Mukhopadhyay S, Talukdar A, Mitra PK, Ghosh S, Maji D. An observational study of the pattern of HIV infection in a specified rural area of India with special reference to migratory laborers. Journal of the International Association of Physicians in AIDS Care. 2010 Mar;9(2):74-7.

4- Azuonwu O, Erhabor O, Obire O. HIV among military personnel in the Niger Delta of Nigeria. Journal of community health. 2012 Feb; 37:25-31.

5- Kapesa A, Basinda N, Nyanza EC, Mushi MF, Jahanpour O, Ngallaba SE. Prevalence of HIV infection and uptake of HIV/AIDS services among fisherfolk in landing Islands of Lake Victoria, northwestern Tanzania. BMC health services research. 2018 Dec; 18:1-9.

6- Corbett EL, Makamure B, Cheung YB, Dauya E, Matambo R, Bandason T, Munyati SS, Mason PR, Butterworth AE, Hayes RJ. HIV incidence during a cluster-randomized trial of two strategies providing voluntary counselling and testing at the workplace, Zimbabwe. Aids. 2007 Feb 19;21(4):483-9.

Stigma related to HIV testing (5 articles)

1- Kipp W, Bajenja E, Karamagi E, Tindyebwa D. AIDS-related stigma: perceptions of family caregivers and health volunteers in western Uganda. World health & population. 2007 Apr 1;9(2):5-13.

2- Nambiar D, Rimal RN. Duty and destiny: psychometric properties and correlates of HIV-related stigma among youth NGO workers in Delhi, India. AIDS care. 2012 Nov 1;24(11):1384-91.

3- Machine EM, Ross MW, McCurdy SA. Issues of expressed stigma of HIV/AIDS among professionals in Southern Sudan. Qualitative health research. 2011 Aug;21(8):1041-50.

4- Masquillier C, Wouters E, Sommerland N, Rau A, Engelbrecht M, Kigozi G, van Rensburg AJ. Fighting stigma, promoting care: a study on the use of occupationally based HIV services in the Free State province of South Africa. AIDS care. 2018 Jun 20;30(sup2):16-23.

5- Ballouz T, Gebara N, Rizk N. HIV-related stigma among health-care workers in the MENA region. The Lancet HIV. 2020 May 1;7(5): e311-3.

Evaluating cost of HIV testing and counselling (3 articles)

1- Jamieson L, Johnson LF, Matsimela K, Sande LA, d'Elbée M, Majam M, Johnson C, Chidarikire T, Hatzold K, Terris-Prestholt F, Nichols B. The cost effectiveness and optimal configuration of HIV self-test distribution in South Africa: a model analysis. BMJ global health. 2021 Jul 1;6(Suppl 4): e005598.

2- De Beer I, Chani K, Feeley FG, Rinke de Wit TF, Sweeney-Bindels E, Mulongeni P. Assessing the costs of mobile voluntary counseling and testing at the workplace versus facility based voluntary counseling and testing in Namibia. Rural and remote health. 2015 Dec;15(4):110-20.

3- Mwaura DM. Achieving sustainable Workplace HIV/AIDS Programmes through a phased out catalytic financing model: case of Swedish Workplace HIV/AIDS Programme in sub-Saharan Africa. In Journal of The International Aids Society 2018 Jul 1 (Vol. 21, Pp. 53-53). The Atrium, Southern Gate, Chichester Po19 8sq, W Sussex, England: John Wiley & Sons Ltd.

Mandatory HIV testing and counselling (3 articles)

1- Egan DJ, Cowan E, Fitzpatrick L, Savitsky L, Kushner J, Calderon Y, Agins BD. Legislated human immunodeficiency virus testing in New York State emergency departments: reported

experience from emergency department providers. AIDS patient care and STDs. 2014 Feb 1;28(2):91-7.

2- Onadeko MO, Balogun MO, Onigbogi OO, Omokhodion FO. Occupational exposure, attitude to HIV-positive patients and uptake of HIV counselling and testing among health care workers in a tertiary hospital in Nigeria. SAHARA-J: Journal of Social Aspects of HIV/AIDS. 2017;14(1):193-201.

3- Panlilio AL, Cardo DM, Grohskopf LA, Heneine W, Ross CS. Updated US Public Health Service guidelines for the management of occupational exposures to HIV and recommendations for postexposure prophylaxis. MMWR Recomm Rep. 2005 Sep 30;54(RR-9):1-7.

Vaccination related articles (2 articles)

1- Vera JH, Hill SC, Rubinstein L. Bacille Calmette-Guerin disease following Bacille Calmette-Guerin vaccination of an HIV-infected health-care worker. International journal of STD & AIDS. 2012 Jul;23(7):1-2.

2- Bakari M, Munseri P, Francis J, Aris E, Moshiro C, Siyame D, Janabi M, Ngatoluwa M, Aboud S, Lyamuya E, Sandström E. Experiences on recruitment and retention of volunteers in the first HIV vaccine trial in Dar es Salam, Tanzania-the phase I/II HIVIS 03 trial. BMC Public Health. 2013 Dec; 13:1-8.

Focusing on HIV treatment (2 articles)

1- George G. Workplace ART programmes: Why do companies invest in them and are they working? African Journal of AIDS research. 2006 Sep 1;5(2):179-88.

2- Meyer-Rath G, Pienaar J, Brink B, van Zyl A, Muirhead D, Grant A, Churchyard G, Watts C, Vickerman P. Company-level ART provision to employees is cost saving: a modelled cost-benefit analysis of the impact of HIV and antiretroviral treatment in a mining workforce in South Africa. In Journal of The International Aids Society 2012 Oct 1 (Vol. 15, Pp. 274-274). Avenue De France 23, Geneva, 1202, Switzerland: Int Aids Society.

Ongoing study protocol (1 article)

1- Muwanguzi PA, Ngabirano TD, Kiwanuka N, Nelson LE, Nasuuna EM, Osingada CP, Nabunya R, Nakanjako D, Sewankambo NK. The effects of workplace-based HIV self-testing on uptake of testing and linkage to HIV care or prevention by men in Uganda (WISe-Men): Protocol for a cluster randomized trial. JMIR Research Protocols. 2021 Nov 1;10(11): e25099.

VCT service evaluation (1 article)

1- Ginwalla SK, Grant AD, Day JH, Dlova TW, Macintyre S, Baggaley R, Churchyard GJ. Use of UNAIDS tools to evaluate HIV voluntary counselling and testing services for mineworkers in South Africa. AIDS care. 2002 Oct 1;14(5):707-26.
